# (How) do medical students regulate their emotions?

**DOI:** 10.1186/s12909-016-0832-9

**Published:** 2016-12-12

**Authors:** Karolina Doulougeri, Efharis Panagopoulou, Anthony Montgomery

**Affiliations:** 1 Department of Educational & Social Policy, University of Macedonia, School of Social Sciences, Humanities and Arts, Egnatia Street 156, Thessaloniki, 54006 Greece; 2 Aristotle University of Thessaloniki, Faculty of Health Sciences of Aristotle University of Thessaloniki, School of Medicine, Thessaloniki, Greece

**Keywords:** Medical students, Critical incidents, Emotions, Emotion regulation

## Abstract

**Background:**

Medical training can be a challenging and emotionally intense period for medical students. However the emotions experienced by medical students in the face of challenging situations and the emotion regulation strategies they use remains relatively unexplored. The aim of the present study was to explore the emotions elicited by memorable incidents reported by medical students and the associated emotion regulation strategies.

**Methods:**

Peer interviewing was used to collect medical students’ memorable incidents. Medical students at both preclinical and clinical stage of medical school were eligible to participate. In total 104 medical students provided memorable incidents. Only 54 narratives included references to emotions and emotion regulation and thus were further analyzed.

**Results:**

The narratives of 47 clinical and 7 preclinical students were further analyzed for their references to emotions and emotion regulation strategies. Forty seven out of 54 incidents described a negative incident associated with negative emotions. The most frequently mentioned emotion was shock and surprise followed by feelings of embarrassment, sadness, anger and tension or anxiety. The most frequent reaction was inaction often associated with emotion regulation strategies such as distraction, focusing on a task, suppression of emotions and reappraisal. When students witnessed mistreatment or disrespect exhibited towards patients, the regulation strategy used involved focusing and comforting the patient.

**Conclusions:**

The present study sheds light on the strategies medical students use to deal with intense negative emotions. The vast majority reported inaction in the face of a challenging situation and the use of more subtle strategies to deal with the emotional impact of the incident.

**Electronic supplementary material:**

The online version of this article (doi:10.1186/s12909-016-0832-9) contains supplementary material, which is available to authorized users.

## Background

### Emotions elicited by clinical practice

Medical students are exposed to emotionally intense incidents that can be either negative (i.e. patient suffering, death, breaches of patient safety) or positive (i.e., positive role models exhibiting compassion, empathy, patient-centered behaviors) [1–6].

During medical education, students report emotional problems such as anxiety, depression, burnout or even symptoms resembling post-traumatic stress disorder [7–9]. Previous studies have also shown that students experience a decline in empathy and an increase in cynicism during the course of medical school, leading to compromised patient care and safety as well as increased dropout rates from medical school [10, 11].

However relatively few studies have focused on how medical students’ regulate their emotions in response to emotional incidents [12]. It is crucial to understand the strategies medical students employ to regulate the emotions they experience during training.

### Emotion regulation

Emotion regulation (ER) includes a variety of strategies that a person can adopt in order to manage a certain emotion. Gross outlines five emotion regulatory processes, which can each encompass a multitude of distinct regulatory strategies: situation selection (taking action and actively selecting a situation, where preferred emotions will be most likely to occur), situation modification (modifying a situation to alter its emotional impact), attention deployment (redirecting attention within a given situation), cognitive change (after the occurrence of a situation, changing the appraisals regarding it, in a way that alters its emotional significance), and response modulation (influencing and modifying physiological, emotional or behavioral responses directly) [13–16].

Successful emotion regulation has been shown to contribute to healthcare workers’ performance and well-being; influencing time spent in listening to patients, reducing burnout and increasing pleasurable emotions [17].

Successful emotion regulation can also have an impact on the level of empathy that medical students will experience. If a patient’s distress causes negative emotions in a healthcare worker that she cannot regulate, that carer will have difficulty experiencing empathy for the patient [18, 19].

Taking all this into account, the aim of the present study was to explore the emotions elicited by critical incidents reported by medical students and the associated emotion regulation strategies. Critical incident reports are short narrative accounts used in medical education. They represent short narratives of incidents that medical students judge to be important learning experiences. They present us with a “window” into the formal and informal messages transmitted via healthcare. Critical and/or memorable incidents represent the point at which an organization reveals important information as to what attitudes and behaviors are valorized [20].

## Methods

### Sample and procedure

A convenience sampling was used to recruit medical students to share a memorable incidents occurred during medical training. The entire medical student body from a Medical school in Northern Greece was eligible to participate in the present study. Medical students of both preclinical and clinical years were invited to participate via announcements in the university online platforms. Interested students contacted the research team to arrange an appointment for the interview.

### Training the interviewers- peer interviewing

Our previous experience with qualitative studies with medical students suggested that medical students might be reluctant to share a critical or memorable incident to someone outside of the medical school or someone involved in the formal medical curriculum, especially if the incident was regarded as negative. For this reason, we decided that engaging other medical students to act as interviewers would be less confrontational for participants, enabling them to share possible disturbing emotions and experiences [21].

Medical students participating in an elective course of Communication skills were invited to act as interviewers. The course is an elective for both pre- clinical and clinical students. Participation in the study as interviewer was voluntary and it was not associated with any incentive. EP, an experienced researcher and professor in communication skills, provided the interviewers with a training workshop (1 h 30 min) including theoretical and practical elements of qualitative interviewing for research purposes, where the students practiced with each other, received feedback from their peers and then reflected on the process. In this workshop they were introduced to the principles of qualitative interviewing as well as ethics in research and they were provided with relevant reading material.

In total 50 (from a class of 93) students volunteered to be interviewers and received the training. Among the interviewers 35 were female students and 15 were male students; 13 were preclinical students and 37 were clinical students. Taking into account the number of participants included in the study every interviewer conducted approximately two interviews. The assignment of interviewers and students was quasi-random and availability of time of both parties was a factor in matching the interviewers and students.

### Data collection

An interview guide was developed to facilitate the peer interviewers in the process of data collection (Additional file 1). In every interview participants were asked to describe a memorable incident that occurred during their studies, without suggesting whether it had to be a positive or a negative one. The initial prompt was to ask students to “describe a memorable incident that occurred during their studies”. Interviewers were trained to ask extra questions regarding the setting (e.g., where did the incident take place? who was involved? if the student was not mentioning this information in his/her narrative). In order to assess emotions and emotion regulation, interviewers asked students about their reactions after encountering the incidents as well as the reactions of others. Students were not asked directly about emotions and regulation strategies but the more generic questions, such as: how did you react after seeing this? And did you do anything/ did anybody do something? Finally students were asked to reflect back on this event as a total experience and make a reflection about thoughts and emotions associated with the event; “Looking back on the event, what are your thoughts, emotions, about it”. This final prompt taps into any emotions and associated emotion regulation that have not been reported yet. All answers were audio recorded.

In total 104 students consented to be audio recorded. Interviewers assigned a special code to each audio file, which contained the interview. After completing the interview, the peer interviewers gave the recorded audio file to a researcher (KD).

### Data analysis

The first author, who does not work in the medical school and has sole access to the audio recordings, transcribed verbatim all interviews and together with EP analyzed the data. All narratives were initially coded based on whether they included explicit or implicit reference to an emotion. Explicit reference to an emotion was made through the use of words referring to emotions (e.g. I felt sad, shocked, angry, happy etc.), while implicit reference concerned cases where the students did not describe explicitly the associated emotion but it was implied by nonverbal cues (e.g. tone of voice, crying). Fieldnotes were not gathered by the interviewers. Emotions were then firstly categorized broadly into two large categories, positives and negatives and subsequently negatives were further divided in more categories. Then, narratives were coded for any reference to strategies aimed at regulating the associated emotion during or after the incident. We followed the categories based on Gross’ model of Emotional Regulation; however, we developed our own themes with regard to specific ER strategies identified in each category. After reading independently the first 20 incidents, saturation of themes had already emerged. The two first researchers rated independently all incidents and then compared their coding. Interater reliability was 95%. Disagreements were resolved by the input of a third researcher (AM) and after rereading carefully the transcripts a consensus was reached.

## Results

### Demographic characteristics of participants

The sample included 54 medical students’ memorable incidents. Among those students, 35 were female and 19 were male. Seven students were preclinical, 2 of them in the 2^nd^ year of studies and 5 in the 3^rd^ year. Forty seven students were in clinical years and among them 34 were in the 4^th^ year, 9 of them were in the 5^th^ year and 5 of them were in the 6^th^ year of medical school.

### Types of incidents, emotions and ER strategies

52.9% of the total sample of memorable incidents included references to emotional incidents (*n* = 54) and only those narratives that included references to emotions were included in the analysis.

In total 66 references to emotions and 47 references to ER strategies were identified in the memorable incidents. Table 1, provides a synopsis of the themes identified in the incidents as well as the categories of emotions and ER strategies.

**Table 1:** Synopsis of findings

Stage 1	Stage 2		
104 medical students provided memorable incidents	54 out of 104 medical students provided experiences with emotions and/ or ER strategies		
Themes identified:	Themes identified	Emotions identified	ER strategies
**1. First contact with:**	**1. First contact with:**	**Positive emotions**	• Inaction (doing/saying nothing) • Trying to comfort the patient • Challenging/trying to change the situation • Focusing on task • Denial • Reappraisal • Distancing oneself from the situation • Suppression
a patient suffering a patient with a terminal disease a patient dying	a patient suffering a patient with a terminal disease a patient dying	• Happiness • Joy • Admiration **Negative emotions** • Shock, bewilderment and surprise • Embarrassment, shame, feeling unease or uncomfortable • Anger • Stress, tension • Sadness or pity • Implied	
**2. Interactions**	**2. Interactions**		
interactions D-P^a^ interactions D-S^a^ interactions D-S^a^ interactions D-D^a^	interactions D-P interactions D-S interactions D-S interactions D-D		
**3. Clinical incidents**	**3. Clinical incidents**		
performing a clinical task see an interesting clinical case	performing a clinical task see an interesting clinical case		

^a^interactions between doctors and patients (D-P); students and patients (S-P), doctors and students (D-S), doctors with doctors (D-D)

The incidents narrated by the medical students fitted into three broad categories; first contact with patients; interactions with patients and doctors, and clinical incidents (performing a clinical task or see a new interesting case). The same categories were identified in both incidents including references to emotions and those without emotional references which were not included in the present study.

Table 2 summarizes the type of incidents associated with emotions as explained in detail below.

**Table 2:** Types of incidents associated with emotions

Types of incidents	Emotions								
	Embarrassment, shame, unease	Shock surprise	Admiration	Sadness pity	Stress	Implied	Anger	Positive	Total
First contact with patient dying	0	5	0	0	0	0	0	1	6
First contact with patient suffering	1	2	0	2	0	0	0	0	5
First contact with patient with terminal disease	0	1	0	0	0	0	0	0	1
Interaction D-P^a^	2	6	1	4	2	8	1	2	26
Interaction S-P^a^	0	3	0	0	0	1	0	0	4
Interaction D-S^a^	0	2	1	0	0	2	1	0	6
Interaction D-D^a^	1	0	0	0	1	0	0	0	2
Performing a clinical task	0	0	0	0	1	0	1	0	2
Interesting clinical case	0	1	0	1	0	0	0	0	2
Total	4	20	2	7	4	11	3	3	54

^a^ *D-P* interactions between doctors and patients, *S-P* students and patients, *D-S* doctors and students, *D-D* doctors with doctors

### Emotions reported by medical students

Table 3 provides examples of excerpts associated with emotions, presented by medical students.

**Table 3:** Types of emotions found in students’ memorable incidents and some exemplary excerpts

Emotion	Excerpt
Happiness	*When the doctor succeeded to reanimate the patient and she [the patient] opened her eyes, I felt so much happiness and gratitude I had seen this (101, female student, 4* ^*th*^ *year)*
Admiration	*The doctor despite the urgent situation did not lose his composure and he seemed very confident. I admired his attitude at that moment. (101, female student, 4* ^*th*^ *year)*
Shock, bewilderment and surprise	*I was shocked of course because it was the first time I saw a decapitated leg which was bleeding (021, female student, 4* ^*th*^ *year)* *I was shocked, the man [the patient] had a green color, his wife was shouting and crying, the doctors were trying to reanimate him and I was just standing there as a statue, unable to believe that I was seeing a person dying in front of me (104, female students, 4* ^*th*^ *year)*
Embarrassment, shame, feeling unease or uncomfortable	*I felt really embarrassed, she [the professor] humiliated me in front of my colleagues and the patients making me look like a completely ignorant (014, female student, 3* ^*rd*^ *year)* *The professor asked me in front of my colleagues and four patients a question and because I asked him to repeat it, he started shouting and wondering how I managed to enter the Medical School. He humiliated me in front of so many people and then he left me standing there. I felt really embarrassed (085, female student, 5* ^*th*^ *year)*
Sadness or pity	*It was the first time seeing a patient in such a critical condition and that made me feel sad about him. How unexpected life can be for some people (004, female student, 6* ^*th*^ *year)* *The doctor at the beginning of the consultation was quite polite with the patient and her husband…but at some point the husband was asking so many questions and the doctor seemed to be in a hurry and he made a sarcastic comment about him. The husband immediately stopped asking and he was obviously embarrassed. I did not like this behavior I felt sad and sorry for the man (017, female student, 3* ^*rd*^ *year)*
Stress, tension or being in hurry	*I have learned that we have to introduce ourselves and say hello to the patient but the attending physician told me, Let’s see if you succeed to complete all your history taking if you continue socializing with the patients. These things are nice but also a luxury when the time is short. I felt weird with this comment, I felt stressed to do things in a way that was superficial (011, female student, 3* ^*rd*^ *year)* *One night in the emergency room they brought a person after a serious car accident. Everybody was in panic and I was also very stressed because it was the first time I encountered such an incident (047, female student, 4* ^*th*^ *year)*
Anger	*That event still makes me angry, because I let a patient be humiliated in front of me (009, female student, 4* ^*th*^ *year)* *The doctor during the surgery was quite rude towards the residents, using really bad language, and I could not understand why he was behaving like this…initially I felt sad but at the same time I was also angry (020, female student, 4* ^*th*^ *year)*

### Positive emotions associated with reported incidents

Positive emotions were reported in 7 cases. Four incidents had to do with feelings such as happiness and gratitude and 3 incidents evoked feelings of admiration for someone else (attending physician or resident). Feelings of happiness resulted from an emergent situation where the doctors successfully helped the patient encounter end well. Words such as “happy”, “satisfied”, “blessed”, “grateful” were used by medical students to describe their emotions related to the incidents.

### Negative emotions associated with reported incidents

Negative emotions were reported in 45 cases. Those negative emotions were divided in the following categories: (1) shock, bewilderment and surprise; (2) feeling embarrassment, shame, unease or uncomfortable; (3) feeling sadness or pity; (4) feeling stress, tension and (5) feeling anger.

### Multiple escalating or coexisting emotions

In 42 out of 54 incidents reported by the participants, there was one predominant emotion reported throughout the incident. However, in 8 incidents we found either escalation of emotion belonging in the same category of emotions (for example, initial feelings of bewilderment followed by feelings of shock) or multiple coexisting emotions belonging in different categories arising from the same incident. In these incidents, the sequences of emotions reported were: feeling unease followed by feeling sad; feeling shocked, feeling sad and finally feeling angry; feeling embarrassed and feeling shocked, feeling shocked followed by sadness; feeling shocked followed by feeling angry; feeling surprised followed by feeling gratitude.

#### Shock, bewilderment and surprise

Feelings of shock, bewilderment and surprise were reported in 25 incidents. Students reported shock when it was the first time encountering a behavior or a clinical case for which they were not emotionally prepared. Incidents of uncivil, rude behavior towards patients initiated by healthcare professional were also related to feelings of shock.

#### Embarrassment, shame, feeling unease or uncomfortable

Personal incidents of humiliation in front of patients or colleagues were reported and provoked negative emotions to students who felt powerless to defend themselves and thus they felt exposed and embarrassed in front of others. Incidents of breaches of patient dignity were also associated with feelings of discomfort and shame reported by students.

#### Sadness or pity

Incidents that had to do with personal contact with patients or observed patient doctor interactions elicited feelings of sadness and pity.

#### Stress, tension

Many students described the hospital as a very busy place where everything happens fast. They also reported observing healthcare professionals to be “in a permanent state of emergency”. Students also reported a feeling of urgency to do things in a rushed way.

#### Anger

Reported feelings of anger had to do with observed interaction between doctor and patients and were reported in cases of observing the compromise of patient dignity.

### ER regulation strategies

With regard to negative emotions, following the model of Gross [13], regulation strategies were categorized into: (1) situation modification strategies, (2) attention deployment strategies, (3) cognitive change strategies and (4) response modulation strategies. Table 4 summarizes all types of ER strategies identified in each category. All types of ER strategies are analyzed in detail below. Table 5 provides short excerpts associated with each type of ER.

**Table 4:** ER strategies used by medical students following Gross taxonomy

Emotion	Situation modification	Attention Deployment	Cognitive change	Response Modulation
Shock, bewilderment, surprise Shame, embarrassment Sadness, pity Anger Anxiety, stress, tension	Inaction (doing/saying nothing) Trying to comfort the patient Challenging/trying to change the situation	Focusing on a task	Denial Reappraisal: Putting emphasis in clinical competencies of doctor Reappraisal: Justifying doctor’s behavior	Suppression of emotions Discussing with peers

**Table 5:** ER strategies and excerpts from participants’ narratives of incidents

Inaction	*A mother and her son arrived in the emergency department and the boy had a fracture in his arm. He was in pain and he was crying. I tried to ask a resident to look after the boy but the answer was “I have more serious things now, they can wait”. I was shocked for the answer but I couldn’t do anything more (065, male student, 3* ^*rd*^ *year)*
Trying to comfort the patient	*The doctor was explaining the use of an external pacemaker- which was supporting a patient after the surgery- While the patient was conscious and could listen to what the doctor was explaining to us- the doctor removed the battery of the external pacemaker for a few seconds- making a joke that the battery is crucial for the survival of the patient. Then he left the patient uncovered and as a result the patient started trembling from cold. I was shocked from the behavior of this doctor who was irresponsible and inhuman. They only thing we could do was to cover the patient in order for her not to feel cold” (037, female student, 4th year)*
Challenging the situation	*[while a doctor was trying to convince a patient to undergo the same medical procedure for teaching purposes and the patient looked really uncomfortable]…We were all feeling awkward so my colleague asked directly the doctor: do you think what you do is appropriate? (010, male student, 4th year)*
Focusing on a task as a way to avoid the emotion	*The doctor had an argument with the wife of the patient but it happened that I was also there to take the history. I was listening to the argument and I was feeling really uncomfortable being there and seeing how sad the patient was but I had to complete my task and leave (036, female student, 4th year)*
Denial	*I didn’t want to believe that the doctor was listening to the patient screaming from pain, and he was ignoring her and continuing the lesson (061, female student, 4* ^*th*^ *year)* *I could not believe the doctor made such an inappropriate comment for the patient, I pretend I did not hear it* *(032, female student, 4* ^*th*^ *year)*
Reappraisal: Justifying unprofessional behavior	*I thought that the doctor was tired or stressed or maybe something had happened before between the patient and himself and taht it why he behaved like this (006, female student 4* ^*th*^ *year)*
Reappraisal: Putting emphasis on clinical competencies of a doctor	*I was very shocked with his [consultant] behavior towards the patient, because he is very good at his job (086, female student, 5* ^*th*^ *year)*
Suppression	*I remembered a time when I had to take history from a patient, who was in a quite serious condition. When I approached her, I introduced myself and I explained the reason I was there and I asked her the reason she was in the hospital. She answered to me: “what do you want to know? I tried to commit suicide” I remembered I was shocked, I did not know what to say. I tried to remain calm, to be emotionally neutral and not show my bewilderment. I continued the history taking as usual but I continued thinking of it (057, male student, 4* ^*th*^ *year)*
Discussing with peers	*When the incident happened, I did not do anything, but I know all my fellow students thought the same as me and we commented on the doctor’s behavior after the lesson (001, female student, 4* ^*th*^ *year)*

### Situation modification

When students experienced an intense negative emotion which was directed either to them, a fellow student or a patient, medical students had the option either to remain passive and inactive or actively try to change the situation.

#### Inaction

Inaction or perceived inability to react was the most frequent response when medical students were facing a negative emotion directed either to them or to a patient. Their perceived inaction was most often associated with suppression of emotions. Medical students chose inaction for two main reasons: perceived lack of knowledge and/or skills and due to low position in hierarchy.

#### Trying to comfort the patient

When a patient was the victim or recipient of an uncivil behavior, medical students felt unable to defend the patient and challenge the doctor. Instead, as a way of regulating emotions, they showed increased affection to the patient.

#### Challenging the situation

On a number of occasions students actively challenged a situation that was generating negative emotions. For example in two cases the student questioned the approach or the behavior of the doctor towards the patient.

### Attention deployment

Attention deployment involves directing one’s attention towards or away from an emotional situation. In this category of ER strategies, only one type of this regulation was identified, shifting attention from the emotion to a task to be accomplished.

#### Focusing on a task as a way to avoid the emotion

Some students in the face of a challenging situation tried to focus their attention on the task they had to accomplish. In that way they tried to avoid regulating the emotion.

### Cognitive change

Cognitive change involves changing how one appraises a situation so as to alter its emotional meaning. Three types of cognitive change were identified: denial and two types of reappraisal.

#### Denial

Some students in the face of an intense incident denied that the doctor was intentionally exhibiting unprofessional behavior. Denial was always associated either with an effort to justify doctor’s behavior by emphasizing clinical competencies.

#### Reappraisal: justifying unprofessional behavior

In cases where students encountered an unprofessional behavior exhibited by a doctor, they tried to justify his/ her behavior but attributing the unprofessional behavior to external circumstances, such as workload, fatigue or time pressure.

#### Reappraisal: putting emphasis on clinical competencies of a doctor

In the face of unprofessional behaviors exhibited by a senior doctor some students tried to emphasize other competencies (e.g., good clinical diagnosis skills) as a way to justify the rude behavior.

### Response modulation

#### Suppression

Expressive suppression is an example of response modulation and involves inhibiting emotional expressions. There were cases where the student despite feeling emotional had to suppress the feeling as a way to be more efficient in the task that they had to perform. Medical students realized that when encountering patients or other healthcare professionals negative feelings may arise but it is necessary to control them in order to complete successfully the requested clinical tasks.

Suppression was frequently associated with inaction in the face of a challenging situation; respondents not only remained passive but they actively tried to hide their emotions.

#### Discussing with peers

Discussing with peers was also reported as a way to find explanations and reflect on the meaning of the behaviors that resulted in intense emotions.

### The aftermath of emotion regulation

In cases where ER included suppression some students reported the impact of the regulation. Some students reported engaging in ruminative thinking or regretting not taking action to defend the patient or themselves. Some students reported that after encountering a stressful incident they chose to leave the clinic and make an effort not to be informed about the patient anymore.

Finally, some students commented on the need to dissociate from feelings in order to be able either to take better decisions or avoid being affected by the human pain encountered in clinical practice.

## Discussion

The present study explored medical students’ emotions elicited by memorable incidents and the associated ER strategies. Medical students’ incidents provided an insight in the type of incidents they consider memorable as well as, the emotions associated with them and the regulation strategies used to mitigate the effect of emotions. Students engage in a variety of ER strategies when experience a challenging situation and the way they decide to handle their emotions can have a significant impact on their personal and professional development [12, 22].

Overall, our students chose to describe emotionally intense negative incidents. Issues related to suboptimal patient care, hierarchy, respect, mistreatment were identified and were in accordance with previous studies [3, 12, 23, 24].

Even though exposure to unprofessional behaviors has been studied before, emotions related to those incidents remain a relatively unexplored topic. In the present study, only 53% of the medical students’ incidents included references to emotions. Among the 54 incidents including emotions, the majority of them (87.28%) referred to negative emotions. The most prevalent emotion in the narratives was shock in the face of unexpected situations such as a patient in a serious condition or observing breaches of patient dignity. Previous studies exploring emotions in the face of professionalism dilemmas or students written reflections about professionalism reported mixed results. For example, Karnieli-Miller and colleagues [23] identified few emotions expressed in written narratives about professionalism whereas a series of studies by Rees, Monrouxe and colleagues [2–4, 12] using oral narratives revealed the existence of many negative emotions associated with the students’ narratives. The latter research, with more expressed emotion, is due to the fact that students narrated their incidents instead of writing about them. Even though in the present study students narrated their incidents instead of writing about them, we found emotions only in about half of the incidents.

One possible explanation can be the one suggested by Karnielli- Miller and concerns the norms in medical education, which are against acknowledging or displaying emotions [23].

Gaufberg and colleagues [24] in their study regarding the hidden curriculum of medical education, reported that medical students in their reflections described the need to actively suppress emotions in response to the powerful incidents of hospital life; other students wrote of distracting or disassociating oneself from “normal” emotional responses to suffering and death, and still other students wondered about their own *lack* of emotions.

In this study, we don’t know whether the students not reporting any emotions did not actually experience them, suppress them or whether they had actually successfully regulated them and thus, there was no emotional residual to report.

### ER strategies

Students in our study chose not to act in several situations even though their personal values were into contrast with the attitude or behavior exhibited by the doctor. A low position in the hierarchy and a fear to challenge a senior colleague have been reported as reasons for inaction in students feeling moral distress [22]. Medical culture often emphasizes the hierarchical structure which does not allow lower members in the hierarchy question or challenge the behavior of the perpetrator.

Several studies have reported that in the face of professional dilemmas, students avoid addressing directly the situation and they prefer more subtle ways to express their disagreement [4].

In our study, when students chose to react, it happened by providing support to the recipient of the negative behavior (i.e., patients) as an act of expressing their disagreement. Few of them chose to question doctor’s behaviors or decision but even in those cases, medical students did not really insist in their reaction when they realized that the doctor was not really changing his behavior or attitude.

Suppression has been identified as a common yet maladaptive ER strategy in several demanding professions such as military officers, police officers and judges [25–27]. In the present study, suppression of emotion was often explained by the students as the selected regulation because emotions were hampering effectiveness in performing a task. It is true that emotions consume cognitive resources by focusing the attention of the person on the object of emotion instead of the task [28]. This implies that emotions are requiring part of the cognitive resources aimed for task accomplishment, impacting negatively the performance of the student [29]. In this sense, suppression of emotion might lead to a short term regulation of intense emotion so the student will not lose the control and accomplish successfully the task.

In medical culture, suppression of emotion is a pragmatic short-term survival strategy, the long-term consequences—for doctors, for patients, and for the wider health care system— are potentially grave. Multiple studies demonstrate the increase of depersonalization and burnout as well as the erosion of empathy over the course of medical training possibly, distancing from self leads to distancing from patients [8, 10, 11, 30].

Another regulation employed by medical students was a reappraisal of the stressful situation by justifying bad behavior and focusing on the positive competencies of a doctor**.** Students tended to reduce the emotional intensity of the incident by reappraising the situation and putting emphasis on external circumstances that could be influencing the bad behavior. There is a tendency for medical students to engage in cognitive dissonance – meaning s/he is a ‘good’ doctor just having a bad day. Such attributions are supported by a culture were clinical skills are judged to be more important than interpersonal skills. Thus, the clinical outcome is more important than the ‘bed-side’ manner.

With regard to the impact associated with ER strategies employed, suppressed emotion was related to more intrusive or ruminating thoughts related to the incident and feelings of guilt. Two of them reported that they actively tried to avoid places reminding them of the incident or avoiding the patient who created the intense emotions. The importance of such ruminative and persistent thoughts relate to how they affect ability to recover from work properly [31]. Dissociation of emotions can help medical students, in the short term, to cope with their intense emotions but in the long term it can lead to decreased empathy and cynicism.

Even though the importance of successful emotion regulation in the face of intense negative events has been recognized in the field of healthcare as well as other demanding professions, research with regard to interventions or training for emotion regulation strategies remains limited. Berking et al. [32] developed and piloted a manualized emotion-regulation training (Integrative Training of Emotional Competencies; iTEC) aimed at improving the emotion-regulation skills of police officers. Police officers often encounter challenging situations associated with intense negative emotions and they are likely to use emotion-regulation strategies such as denial, suppression, and overall avoidance of negative emotions [33]. iTEC involves training police officers in several skills involved in emotion regulation such as (a) muscle relaxation; (b) breathing relaxation; (c) nonjudgmental perception of emotions; (d) acceptance and tolerance of emotions; (e) compassionate self-support; (f) identification of the causes of one’s emotional reaction, and (g) active modification of emotions. Berking et al. [32] piloted iTEC in a small sample of police officers and they reported that the training significantly enhanced successful skill application, especially some skills with which officers reported difficulty applying compared to the control group. Intervention and training programs regarding adaptive ER strategies as well as mindful emotion regulation [34, 35] provide useful directions towards developing emotion regulation training programs also for medical students and healthcare professionals.

### Strengths and limitations

The present study employed a peer interviewing methodology as way to facilitate medical students to share their experiences in a less confrontational environment. Evaluating the experiences of medical students acting as interviewers was beyond the scope of the present study, but our overall estimation is that medical students showed genuine interest to the topic of emotions as well as qualitative interviewing. Thus, we believe that peer interviewing can be seen as research capacity building strategy that can introduce the topic of emotion to medical students acting as interviewers as well as those being interviewed.

On other hand, this study has several limitations. Our study included a convenience sample of students sharing memorable incidents occurred during medical training. All students were from the same Medical School so all of them were exposed to similar learning experiences. In addition, given that the match between participants and interviewers occurred based on time availability of both sides we cannot know whether other factors such as gender and stage of training influenced their interaction. Our findings are heavily skewed towards negative incidents; however, it is likely that incidents involving shock, embarrassment or sadness are easier to recall and do not necessarily represent the majority of medical students’ incidents. In addition, almost half of the students participating in the study did not mention any emotions for the same types of incidents that their counterparts made emotional references. We do not know whether those students did not mention emotions because they did not experience them, suppress them or whether they had actually successfully regulated them. Future research should explore the association between experience of intense incidents, emotion regulation strategies and recollection of those experiences.

## Conclusions

Dysfunctional or the absence of emotion regulation can have a detrimental impact on medical students’ motivation, performance, identity development and their overall well- being. It is a risk factor for burnout (i.e., emotional exhaustion, and cynicism) [35]. Future work should aim to explore how different ER strategies are associated with students’ well-being, professional development and performance.

Despite experiencing intense emotions, most of the students in our study chose not to react and/or tried to suppress them. While it is important for a doctor to remain calm in the face of an urgent situation, students would benefit from learning adaptive regulation strategies instead of only suppression (which is a short term approach). Emotions are integral to medical education and instead of expecting students to figure out what is the best way to cope- or not, they should be empowered with the skills to acknowledge, accept, and regulate them.

## Additional file

Additional file 1: Interview guide for peer interviewers. Description of data: an interview guide was developed to facilitate the peer interviewers in conducting the interviews. The guide included general instructions for the interview process, the core questions of the interview, as well as a checklist of important information that peer interviewers should collect at each stage of the interview. (DOCX 15 kb)

## Abbreviations

ER: Emotion regulation

## References

[1] Feudtner C, Christakis DA, Christakis NA (1994). Do clinical clerks suffer ethical erosion? Students’ perceptions of their ethical environment and personal development. Acad Med.

[2] Monrouxe LV, Rees CE, Endacott R, Ternan E (2014). “Even now it makes me angry”: health care students’ professionalism dilemma narratives. Med Educ.

[3] Rees CE, Monrouxe LV, McDonald LA (2015). “My mentor kicked a dying woman”s bed … analysing UK nursing students’ “most memorable” professionalism dilemmas. J Adv Nurs.

[4] Rees CE, Monrouxe LV, McDonald LA (2013). Narrative, emotion and action: analysing “most memorable” professionalism dilemmas. Med Educ.

[5] Branch WTJ, Kern D, Haidet P, Weissmann P, Gracey CF, Mitchell G (2001). Teaching the human dimensions of care in clinical settings. Journal of the American Medical Association.

[6] Haglund MEM, aan het Rot M, Cooper NS, Nestadt PS, Muller D, Southwick SM (2009). Resilience in the third year of medical school: a prospective study of the associations between stressful events occurring during clinical rotations and student well-being. Acad Med.

[7] Tschernig T, Schlaud M, Pabst R (2000). Emotional reactions of medical students to dissecting human bodies: a conceptual approach and its evaluation. Anat Rec.

[8] Dyrbye LN, Thomas MR, Huntington JL, Lawson KL, Novotny PJ, Sloan JA (2006). Personal life events and medical student burnout: a multicenter study. Acad Med.

[9] Rosenthal JM, Okie S (2005). White coat, mood indigo — depression in medical school. N Engl J Med.

[10] Hojat M, Mangione S, Nasca TJ, Rattner S, Erdmann JB, Gonnella JS (2004). An empirical study of decline in empathy in medical school. Med Educ.

[11] Thomas MR, Dyrbye LN, Huntington JL, Lawson KL, Novotny PJ, Sloan JA (2007). How do distress and well-being relate to medical student empathy? A multicenter study. J Gen Intern Med.

[12] Monrouxe LV, Rees CE, Dennis I, Wells SE (2015). Professionalism dilemmas, moral distress and the healthcare student: insights from two online UK-wide questionnaire studies. BMJ Open.

[13] Gross JJ, Thompson RA, Gross James J (2007). Emotion regulation: conceptual foundations. Handbook of emotion regulation.

[14] Gross JJ, John OP (2003). Individual differences in two emotion regulation processes: implications for affect, relationships, and well-being. J Pers Soc Psychol.

[15] Gross JJ. The emerging field of emotion regulation: An integrative review. Rev Gen Psychol. Special Issue: New directions in research on emotion. 1998a;2:271–299.

[16] Gross JJ. Antecedent- and response-focused emotion regulation: divergent consequences for experience, expression, and physiology. J Pers Soc Psychol.1998b;74:224–237. 10.1037//0022-3514.74.1.224 9457784

[17] Zammuner VL, Lotto L, Galli C (2003). Regulation of emotions in the helping professions: nature, antecedents and consequences. Australian e-Journal for the Advancement of Mental Health.

[18] Decety J, Yang C-Y, Cheng Y (2010). Physicians down-regulate their pain empathy response: an event-related brain potential study. Neuroimage.

[19] Gleichgerrcht E, Decety J. The relationship between different facets of empathy, pain perception and compassion fatigue among physicians. Front Behav Neurosci [Internet]. 2014 Jul 11 [cited 2016 Feb 9];8. Available from: http://journal.frontiersin.org/article/10.3389/fnbeh.2014.00243/abstract doi:10.3389/fnbeh.2014.00243 10.3389/fnbeh.2014.00243 PMC4093939 25071495

[20] Brady DW, Corbie-Smith G, Branch J, William T (2002). “What’s important to You?”: the use of narratives to promote self-reflection and to understand the experiences of medical residents. Ann Intern Med.

[21] Byrne E, Brugha R, Clarke E, Lavelle A, McGarvey A (2015). Peer interviewing in medical education research: experiences and perceptions of student interviewers and interviewees. BMC Res Notes.

[22] Wiggleton C, Petrusa E, Loomis K, Tarpley J, Tarpley M, O’Gorman ML (2010). Medical students’ experiences of moral distress: development of a web-based survey. Acad Med.

[23] Karnieli-Miller O, Vu TR, Holtman MC, Clyman SG, Inui TS (2010). Medical students’ professionalism narratives: a window on the informal and hidden curriculum. Acad Med.

[24] Gaufberg EH, Batalden M, Sands R, Bell SK (2010). The hidden curriculum: what can we learn from third-year medical student narrative reflections?. Acad Med.

[25] Tull MT, Barrett HM, McMillan ES, Roemer L (2007). A preliminary investigation of the relationship between emotion regulation difficulties and posttraumatic stress symptoms. Behav Ther.

[26] van Gelderen BR, Bakker AB, Konijn EA, Demerouti E (2011). Daily suppression of discrete emotions during the work of police service workers and criminal investigation officers. Anxiety Stress Coping.

[27] Maroney TA (2011). Emotional regulation and judicial behavior. California Law Review.

[28] Ellis HC, Ashbrook PW, Fiedler K, Forgas J (1988). Resource allocation model of the effects of depressed mood states on memory. Affect, cognition and social behaviour.

[29] Meinhardt J, Pekrun R (2003). Attentional resource allocation to emotional events: an ERP study. Cognit Emot.

[30] Neumann M, Edelhäuser F, Tauschel D, Fischer MR, Wirtz M, Woopen C (2011). Empathy decline and its reasons: a systematic review of studies with medical students and residents. Acad Med..

[31] Fritz C, Sonnentag S (2006). Recovery, well-being, and performance-related outcomes: the role of workload and vacation experiences. J Appl Psychol.

[32] Berking M, Meier C, Wupperman P (2010). Enhancing emotion-regulation skills in police officers: results of a pilot controlled study. Behav Ther.

[33] Amaranto E, Steinberg J, Castellano C, Mitchell R (2003). Police stress interventions. Brief Treatment and Crisis Intervention.

[34] Berking M, Whitley B (2014). Affect regulation training.

[35] Chambers R, Gullone E, Allen NB (2009). Mindful emotion regulation: an integrative review. Clin Psychol Rev.

