# Supplementary material for: (How) do medical students regulate their emotions?
Source: BMC Med Educ. 2016 Dec 12;16:312. doi: 10.1186/s12909-016-0832-9 (PMC5154027; doi:10.1186/s12909-016-0832-9)
Supplement: Additional file 1: — Interview guide for peer interviewers. Description of data: an interview guide was developed to facilitate the peer interviewers in conducting the interviews. The guide included general instructions for the interview process, the core questions of the interview, as well as a checklist of important information that peer interviewers should collect at each stage of the interview. (DOCX 15 kb) [file 12909_2016_832_MOESM1_ESM.docx]

| **Interview guide for assessing critical incidents, emotions and learning in medical students** | | |
| --- | --- | --- |
| General Instructions for peer interviewers:   1. be sure to leave adequate time for the interview 2. check audio recording apparatus and test it 3. welcome the participants 4. explain the purpose of the study and the process of the interview 5. explain confidentiality 6. obtain consent before recording 7. follow the interview guide 8. conclude the interview with care and respect 9. if any issues come up, refer the participant to the study coordinators | | |
|  | Questions | Notes for interviewers |
| 1 | Describe a memorable incident that occurred during your studies | [ ]In case participant asks, the incident can be positive or negative and it could take place at any stage of their studies  [ ]Clarify when the incident took place  [ ] Clarify where the incident took place  [ ] Clarify who was involved |
| 2 | After encountering this incident, what was your reaction? What was the reaction of others? | [ ] Explore feelings associated with the incidents  [ ]Explore the initial reaction of participants [first thoughts, feelings, behaviors] |
| 3 | Did you do anything/ did others do anything? | [ ] Explore how the participant or other people involved in the incident behaved as a response to it |
| 4 | Looking back on the event, what are your thoughts and emotions about it | [ ] If unanswered in previous questions, give a summary of the event and explore emotions and thought associated with it.  [ ]Explore what is the current impression of the participants regarding the incident |
